# Right dorsal colitis in horses: A multicenter retrospective study of 35 cases

**DOI:** 10.1111/jvim.16884

**Published:** 2023-10-06

**Authors:** Jordan Flood, David Byrne, Jennifer Bauquier, Gustavo Ferlini Agne, Jessica C. Wise, Carlos E. Medina‐Torres, Kelly Wood, Olivia Sullivan, Allison J. Stewart

**Affiliations:** ^1^ School of Veterinary Science University of Queensland Gatton Queensland Australia; ^2^ School of Veterinary Medicine Murdoch University Perth Western Australia Australia; ^3^ Melbourne Veterinary School University of Melbourne Melbourne Victoria Australia; ^4^ School of Animal and Veterinary Sciences University of Adelaide Adelaide South Australia Australia; ^5^ School of Animal and Veterinary Sciences Charles Sturt University Wagga Wagga New South Wales Australia; ^6^ Present address: Pferdeklinik SaarLorLux GmbH Ueberherrn Germany; ^7^ Present address: Yarra Ranges Animal Hospital Lilydale Victoria Australia; ^8^ Present address: Scone Equine Hospital Scone New South Wales Australia; ^9^ Present address: NSW Department of Primary Industries Goulburn New South Wales Australia

**Keywords:** colic, diarrhea, nonsteroidal anti‐inflammatory drugs, phenylbutazone

## Abstract

**Background:**

Right dorsal colitis (RDC) is a nonsteroidal anti‐inflammatory drug (NSAID) induced, protein losing enteropathy in horses associated with a high case fatality rate.

**Objectives:**

To describe signalment, NSAID usage, clinical presentations, clinical pathology, ultrasonographic findings, treatments, outcomes, and factors associated with survival in horses diagnosed with RDC.

**Animals:**

Thirty‐five horses from 7 Australian equine hospitals diagnosed with RDC.

**Methods:**

Retrospective case series. Clinical records of cases were accepted if definitively or presumptively diagnosed by an internist with RDC and had ≥3 of: hypoproteinemia or hypoalbuminemia; diarrhea with negative test results for infectious diseases; colic for which other diseases were excluded or right dorsal colon thickening on ultrasound. Descriptive data analysis was performed for categorical and continuous variables. Univariate binominal logistic regressions were used to assess factors associated with survival.

**Results:**

An overdose of NSAIDs occurred in 84% (21/25) cases where dose was known. Common clinical presentations included diarrhea (69%; 22/32), colic (61%; 20/33), and tachycardia (53%, 17/32). Common clinicopathological findings included hypoalbuminemia (83%; 26/31), hypocalcaemia (79%, 23/29), and hyperlactatemia (77%, 14/18). The right dorsal colon wall appeared subjectively thickened in 77% (24/31) cases using ultrasonography. Case fatality rate was 43% (15/35). Odds of survival significantly decreased with increasing heart rate (odds 0.84, 95% CI = 0.71‐0.92, *P* = .01), packed cell volume (odds 0.91, 95% CI 0.82‐0.98, *P* = .05) and abnormal appearance of mucous membranes (odds 0.05, 95% CI 0.005‐0.28, *P* = .001) on hospital presentation.

**Conclusions and Clinical Importance:**

An overdose of NSAIDs is common in horses diagnosed with RDC. Serum albumin concentrations should be monitored in horses receiving a prolonged course of NSAIDs. Overall prognosis for RDC remains fair.

**Abbreviations**
COX: cyclo‐oxygenase
HR: heart rate
IQR: interquartile range
MM: mucous membranes
NSAIDs: nonsteroidal anti‐inflammatory drugs
PCV: packed cell volume
RDC: right dorsal colitis

## 1 INTRODUCTION

Right dorsal colitis (RDC) is a protein‐losing enteropathy localized to the right dorsal colon in horses that can be induced by the administration of nonsteroidal anti‐inflammatory drugs (NSAIDs). It is characterized by marked edema and thickening of the right dorsal colonic walls with ulcerative lesions extending into the mucosa or lamina propria.^1^, ^2^ The disease often occurs subsequent to NSAID usage, particularly with excessive dosages or prolonged administration.^1^, ^2^

Although an exact pathogenesis of RDC remains unclear, it is hypothesized that inhibition of COX enzymes disrupts microvascular endothelium and mucosal barrier function in the right dorsal colon, resulting in thrombosis, infarction, and mucosal ulcerative lesions.^3^, ^4^ Phenylbutazone administration is associated with alterations to blood flow and volatile fatty acid metabolism in the right dorsal colon, which is hypothesized to contribute to the pathogenesis of colonic inflammation and a possible cytotoxic effect on colonic epithelial cells.^4^ There is reduced secretion of bicarbonate in the right dorsal colon in response to administration of phenylbutazone, suggesting that phenylbutazone could inhibit the buffering capacity of the colonic mucosa.^5^ Predilection for lesions in the right dorsal colon is attributable to higher susceptibility to ischemia, slower transit time, and reduced luminal diameter than other sections of the colon, potentially prolonging drug contact time.^1^, ^6^ Few reports of RDC have been published and confirming a presumptive diagnosis of RDC can be challenging with clinical signs similar to other differential diagnoses. Prognosis for RDC is variable, with case fatality rates ranging from 20% to 92%.^1^, ^7^, ^8^, ^9^

To the best of our knowledge, a retrospective study of RDC in equids investigating NSAID usage, certain clinicopathological findings and factors associated with death has not been published, despite extensive use of NSAIDs in horses. The aim of this retrospective study was to describe the signalment, NSAID usage, clinical presentations, clinical pathology, ultrasonographic findings, treatments, outcomes, and factors associated with death of horses diagnosed with RDC.

## 2 MATERIALS AND METHODS

### 2.1 Case selection

A retrospective review of clinical records of RDC cases was performed. Clinical records were solicited from registered specialists in equine internal medicine from private and university equine hospitals in Australia from 2007 to 2021. Cases were included if a definitive diagnosis of RDC was confirmed with biopsy or direct examination of the right dorsal colon via necropsy, laparoscopy or celiotomy. Cases with a presumptive diagnosis of RDC were included if they were diagnosed by an internal medicine specialist, had a history of NSAID use and met ≥3 of the following criteria:, hypoproteinemia or hypoalbuminemia; loose feces/diarrhea with negative results for infectious diseases; colic for which other diseases (eg, sand enteropathy, gastric ulceration) were excluded and right dorsal colon thickening on ultrasound (>3 mm for ponies, >4 mm for horses).^10^

### 2.2 Clinical data

Clinical records for each case were reviewed, and the following information was obtained: signalment (year of admission, age at admission, sex, breed), clinical presentation (clinical signs including mentation, diarrhea and colic), clinical examination variables (including rectal temperature, heart rate, respiration rate, mucous membranes color, capillary refill time), NSAID usage (drug, dosage, duration), clinicopathological results at admission, 24 to 72 hours and 72 to 110 hours after admission, abdominal ultrasonography findings, therapeutic interventions, and outcome.

### 2.3 Statistical analysis

Data from the case series were summarized using standard descriptive statistics. Categorical variables were evaluated using frequency distributions. Continuous data was assessed for normality using the Shapiro‐Wilk test. Normally distributed variables were reported as mean ± SD. Non‐normally distributed variables are reported as median and interquartile ranges (IQR). Statistical analysis for determining factors associated with survival was performed using R statistical software (v4.1.2; R Core Team 2021).

The dataset was highly incomplete. Variables where >60% of the observations were missing were excluded from analysis. The distribution of missing observations in remaining variables was visualized. Given the small number of cases included in the study, the number of missing values, and the likelihood that values for certain observations were not missing completely at random (eg, more clinical examination variables available than clinicopathological variables), multiple imputation by chained equations was applied to all variables with missing values over 10 cycles with an *r* ^2^ > .01.^11^ Univariate binomial logistic regressions were used to assess the association between variables and survival for each imputed data set and results were pooled. Imputation diagnostics were undertaken by comparing the absolute difference in means between observed and imputed values. Statistical significance was set at *P <* .05.

## 3 RESULTS

A total of 39 cases were submitted from 7 institutions for the study. Thirty‐five cases met the inclusion criteria, with 4 excluded due to insufficient clinical data or inability to meet the set criteria. Twelve cases were definitively diagnosed via surgery (4/35), biopsy (1/35), surgery and necropsy (3/35), and necropsy (4/35). Twenty‐three cases were a presumptive diagnosis based on the above set of criteria.

### 3.1 Signalment

Horses that met the inclusion criteria comprised Thoroughbreds (10/35, 29%), ponies (7/35, 20%), Warmbloods (5/35; 14%), Quarter horses (4/35; 11%), Standardbreds (3/35; 9%), draft horses (3/35; 9%), an Arabian (1/35; 3%), an Australian Stock Horse (1/35; 3%), and a horse with an undisclosed breed (1/35; 3%). Geldings accounted for 69% of cases (24/35), with 17% (6/35) mares and 14% (5/35) stallions. The median (IQR) age at time of diagnosis was 6 years (4‐11.5).

### 3.2 NSAID administration

Thirty‐four out of 35 horses were reported to have received at least 1 dose of NSAIDs in the previous 30 days before to hospital admission. One horse did not have details of previous NSAID administration history recorded in the medical record but was definitively diagnosed with RDC at surgery; it is believed by the contributing author that this horse received NSAIDs, however this case was omitted from NSAID administration data in this study due to the unavailability of dosage details. Data for dosage or duration of NSAID administration was available in 25 out of 35 cases. For the 10 horses for which the exact dosage or duration of NSAID administration was unknown, 2 were definitively diagnosed at surgery, 1 definitively diagnosed via biopsy and 1 case definitively diagnosed via necropsy. The remaining 6 cases that did not have the dose or duration of NSAIDs recorded were reported to have recent NSAID administration (treating conditions including chronic laminitis, gastro‐intestinal related‐disease, a laceration and prolonged use for ophthalmic‐related disease) and had a presumptive diagnosis of RDC made based on the above set of criteria.

**FIGURE 1:**
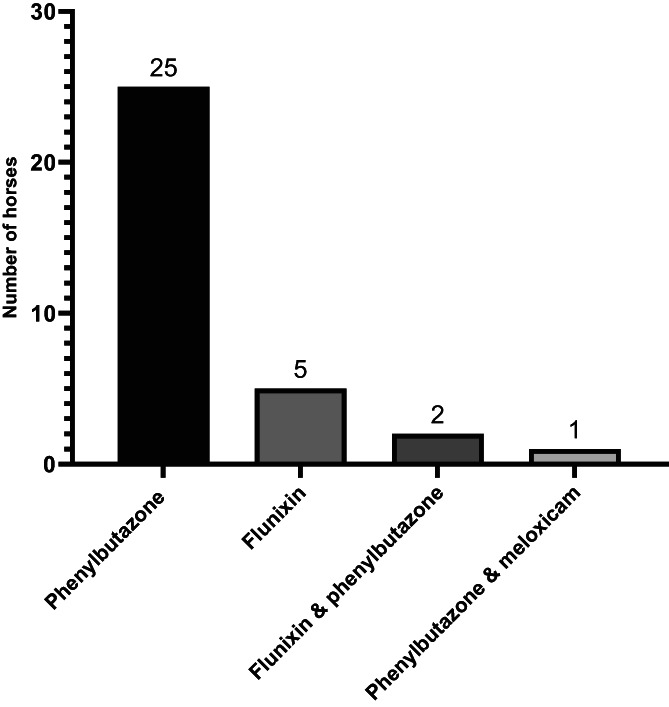
NSAID's administered to cases before diagnosis of RDC.

The most common primary complaint for NSAID administration was nonlaminitis related orthopedic disease (10/34; 29%), followed by laminitis (7/34; 21%). Other reasons for initial NSAID use included ophthalmic‐related disease (6/34; 17%), colic or gastrointestinal related disease (6/34, 17%) and fever (2/34; 6%). Castration was reported as a cause for NSAID administration in 2 cases (6%) and respiratory disease in 1 case (3%). Phenylbutazone was the most commonly administered NSAID (24/34; 74%). Administration of flunixin (5/34; 15%), a combination of phenylbutazone and flunixin (3/34; 9%) and a combination of meloxicam and phenylbutazone (1/34; 3%) was reported (Figure 1).

In cases where total dosage of NSAID administration was known, an overdose of NSAIDs was reported in 84% of cases (21/25). An overdose in this study was defined as receiving >8.8 mg/kg phenylbutazone in a single day, receiving >4.4 mg/kg phenylbutazone within each day for more than 4 days, or receiving >2.2 mg/kg phenylbutazone for more than 8 consecutive days. For flunixin, an overdose was defined as receiving >2.2 mg/kg in a single day. Some cases qualified for more than 1 overdose category in that they received an excessive single dose on 1 day, and received an extended course of NSAIDs over 8 consecutive days. Of the horses that received an overdose (n = 21), 29% of these cases received a dose exceeding 8.8 mg/kg phenylbutazone or 2.2 mg/kg flunixin in a single day. A overdose with horses receiving greater than 4.4 mg/kg phenylbutazone for more than 4 consecutive days occurred in 29% of horses. A total of 76% of horses considered to have an overdose received more than 2.2 mg/kg phenylbutazone for more than 8 consecutive days. The median (IQR) number of days horses received phenylbutazone before adiagnosis of RDC was 9 days (6‐14 days). The median (IQR) total dosage of phenylbutazone (administered as a sole therapy) before a diagnosis of RDC was 58.8 mg/kg (24.8‐75 mg/kg), with the median (IQR) daily dosage being 5.2 mg/kg/day (4‐11 mg/kg/day).

### 3.3 Clinical presentation

Clinical presentation findings at hospital admission are presented in Table 1. The most common clinical presentations were diarrhea (69%; 22/32), colic (61%; 20/33), and tachycardia (53%, 17/32).

**TABLE 1:** Summary of clinical presentation and clinical examination variables of right dorsal colitis cases at hospital admission (n = 35).

Clinical variables at admission	n	Yes	No
Diarrhea	32	22 (69%)	10 (31%)
Colic	33	20 (61%)	13 (39%)
Depression/lethargy	27	15 (56%)	12 (44%)
Tachycardia	32	17 (53%)	15 (47%)
Abnormal mucous membranes	32	16 (50%)	16 (50%)
Tachypnea	28	13 (46%)	15 (54%)
Fever	30	10 (33%)	20 (67%)

Clinical variables at admission	n	Median	IQR
Rectal temperature (°C)	30	38.1	37.7‐38.6
Heart rate (bpm)	32	54	40‐64
Respiratory rate (bpm)	28	22	16‐44
Capillary refill time (seconds)	28	2	1‐2.65

### 3.4 Hematology

Hematology results on hospital presentation were recorded (Table 2). The most common findings were neutrophilia (9/29; 31%), leucocytosis (8/32; 25%), and an increased packed cell volume (PCV; 5/29; 17%).

**TABLE 2:** Hematology and serum biochemistry values of horses with right dorsal colitis at hospital admission (n = 35).

Hematology	N	Median (IQR 25th to 75th percentile)	% cases below reference range	% cases above reference range
RBC (×10^12^/L)	29	8.2 (7.5‐10.9)	13.8 (4/29)	6.9 (2/29)
PCV	29	41 (35‐48)	10.3 (3/29)	17.2 (5/29)
WBC (×10^9^/L)	32	8.7 (6.4‐13.6)	15.6 (5/32)	25.0 (8/32)
Neutrophils (×10^9^/L)	29	5.7 (3.1‐12.8)	13.8 (4/29)	31.0 (9/29)
Lymphocytes (×10^9^/L)	30	2.6 (1.9‐3.8)	16.7 (5/30)	3.3 (1/30)
Monocytes (×10^9^/L)	31	0.4 (0.1‐0.7)	0 (0/29)	12.9 (4/31)
Eosinophils (×10^9^/L)	15	0 (0‐0.09)	0 (0/29)	13.3 (2/15)
Platelets (×10^9^/L)	24	143 (121‐178)	16.7 (4/24)	0 (0/22)

Serum biochemistry	n	Median (IQR)	% cases below reference range	% cases above reference range
Sodium (mmol/L)	29	134 (131‐135)	31.0 (9/29)	0 (0/0)
Potassium (mmol/L)	29	3.5 (3.1‐3.5)	3.4 (1/29)	10.3 (3/29)
Chloride (mmol/L)	23	102 (97.5‐104)	26.1 (6/23)	0 (0/23)
Total calcium (mmol/L)	29	2.5 (2.2‐2.6)	79.3 (23/29)	0 (0/29)
Magnesium (mmol/L)	13	0.6 (0.5‐0.7)	69.2 (9/13)	7.7 (1/13)
Phosphate (mmol/L)	21	1.1 (0.8‐1.7)	42.9 (9/21)	23.8 (5/21)
Total protein (g/L)	30	52 (47.3‐61.8)	50 (15/30)	0 (0/0)
Albumin (g/L)	31	21 (18‐23)	83.4 (26/31)	0 (0/31)
Globulin (g/L)	27	26 (23.4‐31.1)	40.7 (11/27)	3.7 (1/27)
Albumin:globulin ratio	27	0.9 (0.65‐0.95)		
Serum amyloid A	7	683 (937‐1098)	0 (0/0)	100 (7/7)
Creatinine	29	120 (100‐155)	31 (9/29)	24.1 (7/29)
Blood urea nitrogen (mmol/L)	27	7.5 (5.6‐11.4)	7.4 (1/27)	40.7 (11/27)
Creatinine kinase (IU/L)	25	290 (223‐1020)	0 (0/25)	32 (8/25)
AST (IU/L)	26	275 (196.8‐348.3)	30.1 (8/26)	23.1 (6/26)
Total bilirubin (μmol/L)	26	36.1 (21.4‐53.5)	15.4 (4/26)	61.6 (16/26)
Lactate (mmol/L)	18	1.3 (0.8‐2.7)	0 (0/0)	77.8 (14/18)
Fibrinogen (g/L)	25	4 (3‐5.6)	0 (0/22)	44 (11/25)

### 3.5 Serum biochemistry

Biochemistry results on hospital presentation were recorded (Table 2). The most common abnormal clinicopathological findings were hypoalbuminemia (83%; 26/31), hypocalcemia (79%, 23/29), hyperlactatemia (78%, 14/18), hypomagnesemia (69%, 9/13), and an elevated serum amyloid A concentration (100%, 7/7). Hypoproteinemia was only evident in 50% of cases, and hypoglobulinemia was present in 41% cases.

### 3.6 Abdominal ultrasonography

Abdominal ultrasonography was performed in 31 out of the 35 cases. Subjective thickening of the right dorsal colonic wall was reported by the clinician performing the ultrasound in 24/31 cases (77%) and an exact measurement of the mural thickness of the right dorsal colon was recorded in 20 cases. The median mural thickness of the right dorsal colonic wall was 10 mm (7‐13.5 mm).

### 3.7 Therapeutic interventions

Intravenous fluid therapy was reportedly administered in 20/34 cases (59%), misoprostol in 16/31 (51.6%), omeprazole in 15/32 (47%), sucralfate in 12/32 (38%), antimicrobials in 12/32 (37.5%), corn oil and psyllium husks in 5/32 (15.6%), and hyperimmune plasma in 4/32 (13%). A low bulk diet was recommended in 10/26 (39%) cases.

### 3.8 Surgery

Exploratory ventral midline celiotomy was performed in 7/35 (20%) cases, with the large colon partially resected in 2/7 cases. Reasons for performing surgery were documented in 5 cases, all of which reported severe or chronic signs of colic. Of the 2 horses that underwent resection, 1 recorded stricture and marked thickening of the right dorsal colon. Three out of 7 horses (43%) horses that underwent surgery did not survive to discharge. Both horses that had a colon resection survived to discharge.

### 3.9 Outcome

**FIGURE 2:**
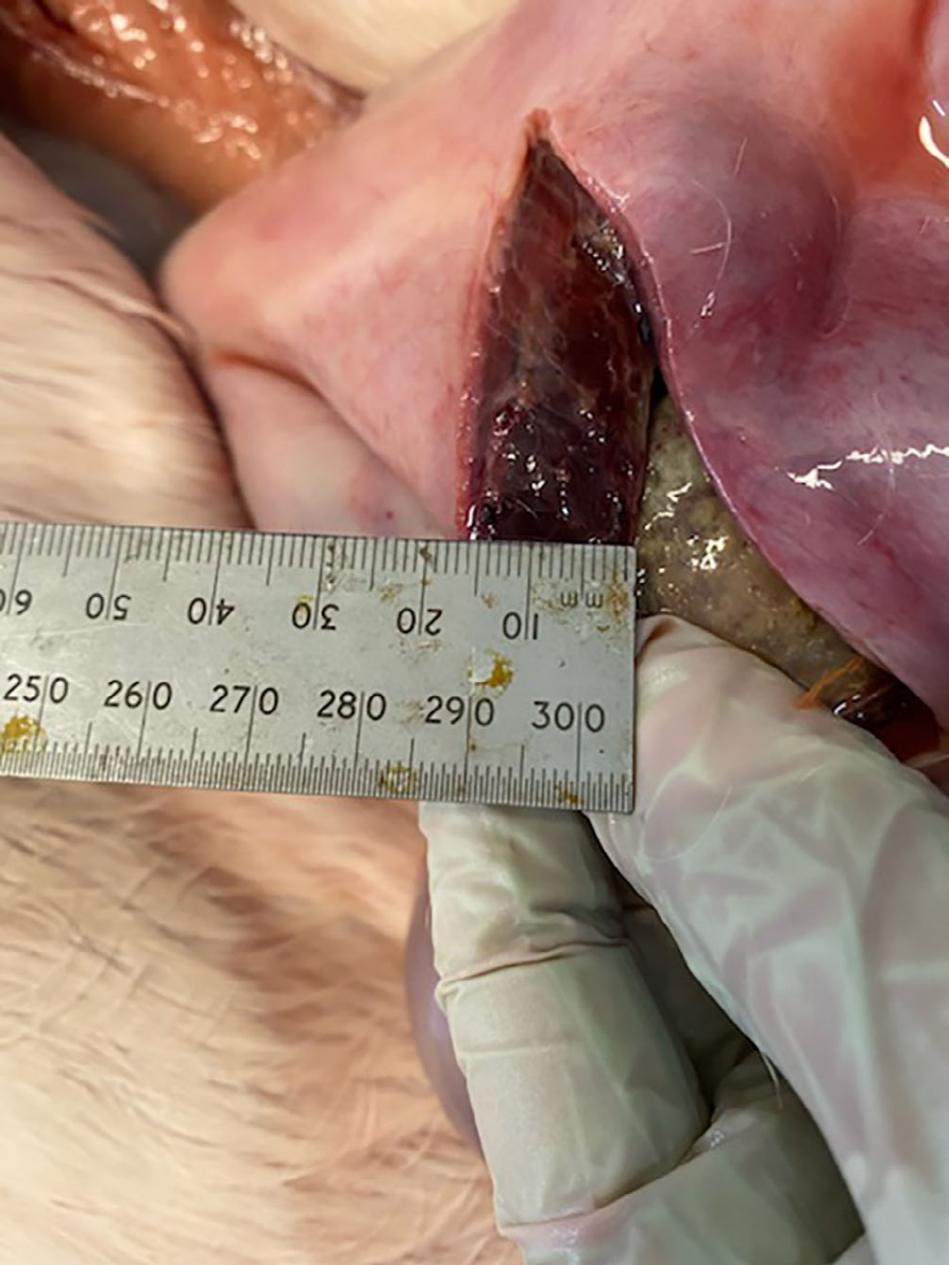
Measurement of right dorsal colon wall thickness at post mortem examination.

**FIGURE 3:**
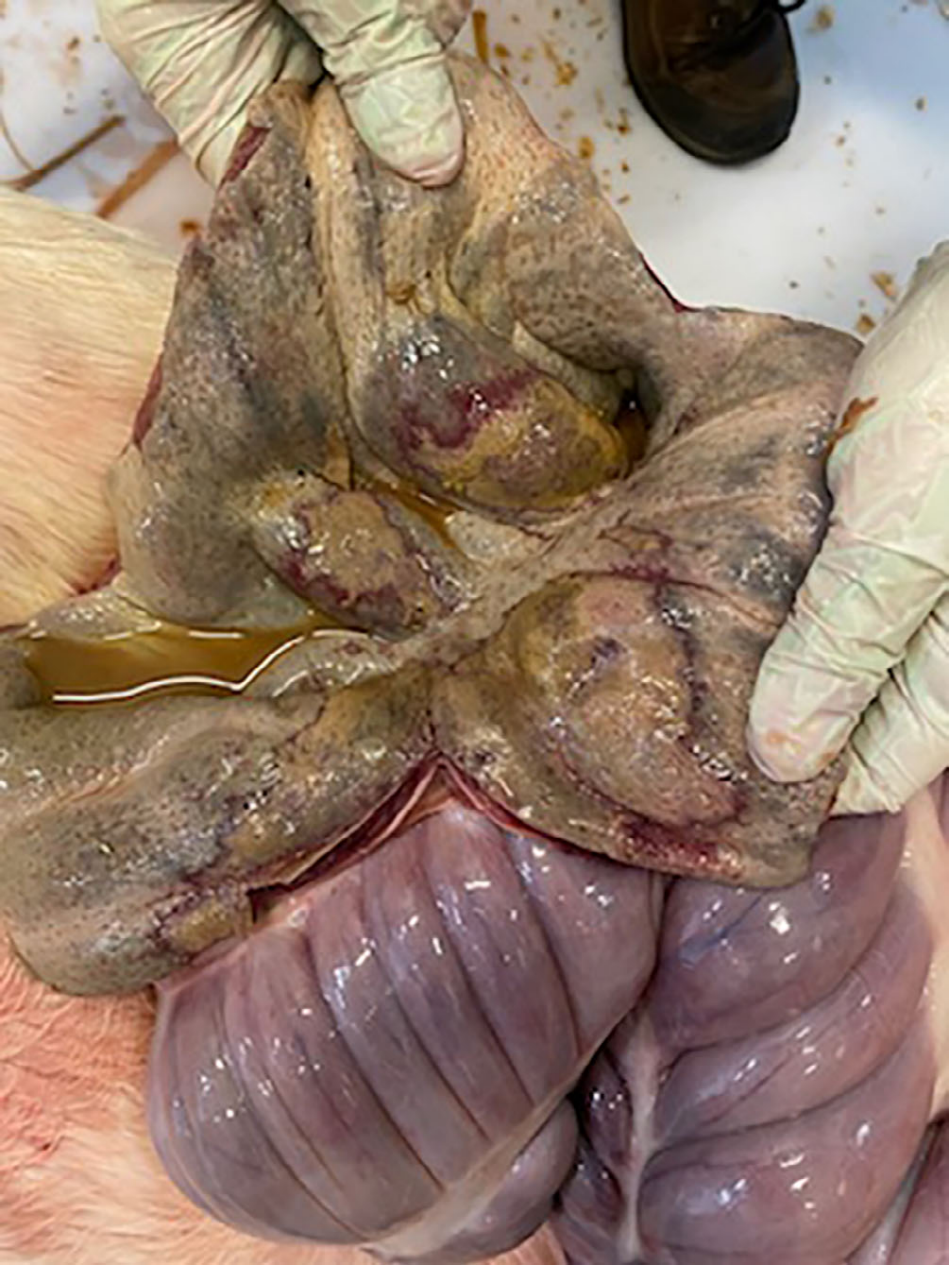
Right dorsal colon on post mortem examination.

The median length of hospitalization was 5 (2–10) days. Survival to hospital discharge was 57% (20/35). Eight horses had a post‐mortem examination performed with ulceration evident in the right dorsal colon of all cases. Common findings reported at post‐mortem included diffuse edema of the right dorsal colon and severe, focally extensive ulcerations (see Figures 2 and 3). One case had concurrent renal papillary necrosis of the right kidney.

### 3.10 Factors associated with survival

Sixty‐six variables were included in the final analysis with 21% of values missing across the data set. Clinical observations (eg, heart rate, respiratory rate, etc) exhibited fewer missing values than laboratory variables (eg, leukogram and biochemistry).

Odds of survival decreased with increasing heart rate (OR 0.84, 95% CI = 0.71‐0.92, *z* = −2.86, *P* = .01) and packed cell volume (OR 0.91, 95% CI = 0.82‐0.98, *z* = −2.15, *P* = .05) on presentation. Horses presenting with abnormal mucous membranes on presentation (OR = 0.05, 95% CI = 0.005‐0.28, *z* = −3.11, *P* = .001) also had a significant decrease in odds of survival. Absolute means of observed and imputed values were compared and found to be <2 SD different. Odds of survival significantly increased with increasing duration of hospitalization (OR = 1.34, 95% CI = 1.10‐1.75, *z* = 2.53, *P* = .05).

## 4 DISCUSSION

This case series describes 35 horses that were definitively or presumptively diagnosed with RDC. The most common clinical presentations included colic and diarrhea. Common clinicopathological abnormalities included hypoalbuminemia, hyperlactatemia, and an increased serum amyloid A concentration. Hypoproteinemia was not as common as reported in the literature, and panhypoproteinemia was not a common finding.^1^, ^7^, ^8^ Transabdominal ultrasonography often revealed thickening of the right dorsal colon wall, however this was not evident in all cases. Odds of survival significantly decreased with an increased heart rate, PCV, and abnormal mucous membranes at hospital presentation. The survival rate in this study was lower than recent reports of RDC, although selection criteria differed among studies.^1^, ^7^, ^8^, ^9^

NSAIDs are one of the most prescribed analgesic and anti‐inflammatory drugs in equine veterinary medicine.^12^ Excessive or prolonged NSAID administration in horses is associated with 3 main adverse effects: gastroduodenal ulceration, renal papillary necrosis, and RDC.^13^ Right dorsal colitis can be difficult to diagnose with vague clinical signs similar to other diseases, such as sand enteropathy, inflammatory bowel disease, and hepatopathies.^6^ Cases were accepted into the study if a definitive diagnosis was made, or a presumptive diagnosis of RDC was made based on history of NSAID use and meeting specific criteria. It is possible that some of the 4 cases that were excluded were true RDC cases but did not have complete medical records or did not meet 3 or more of the criteria to be accepted into the study. It is possible that excluded cases that were positive for infectious pathogens could have been true RDC cases, however this was not investigated in this study. Criteria for a presumptive diagnosis of RDC is variable between other published studies, therefore it is difficult to compare results. Two previous case series only include horses that have been diagnosed via celiotomy or necropsy, which could represent more severe cases of RDC that could not be managed medically.^7^, ^8^ Another study includes presumptive RDC cases in which a history of NSAID usage, ultrasonographic evidence of edema of the RDC and hypoproteinemia are used as criteria; however, it has been suggested that the sensitivity of ultrasonography in RDC cases could be low.^1^, ^14^, ^15^ Our study included 7/31 (23%) cases of RDC that did not have ultrasonographic evidence of right dorsal colon thickening.

While horses of all ages can be affected by RDC, the disease is associated with ponies and younger horses with 95% of RDC cases under 15 years of age.^1^, ^6^ Data from this study is comparable with these findings, with 29/35 cases (82%) under 15 years of age. It is hypothesized that younger horses (under 15 years of age) are predisposed to RDC as they are more likely to develop performance‐associated lameness or musculoskeletal injuries which increases likelihood of being treated with NSAIDs. The most common reason for initial NSAID usage in the current study was nonlaminitic related orthopedic disease, supporting this hypothesis. Ponies and foals are considered predisposed to RDC due to difficulty determining weight and increased risk of excessive dosage of NSAIDs.^6^ Ponies have increased individual sensitivity to NSAID usage in comparison to horses, although this was not investigated within this study.^16^

In this study, 84% of horses where the dose and duration of NSAID administration was known received an overdose before the development of RDC. These results are consistent with previous reports that prolonged administration or excessive dosages of NSAIDs increase the risk of developing RDC.^1^, ^6^, ^16^ Of the horses that received an overdose, 76% had received at least 2.2 mg/kg phenylbutazone for at least 8 consecutive days. This suggests that phenylbutazone might not be safe for prolonged use, and other analgesic drugs such as paracetamol, should be considered for long term use. Phenylbutazone is the predominant NSAID associated with RDC, and other NSAID‐induced lesions.^1^, ^6^ It is unknown whether this is due to a lower safety profile, increased usage in comparison to other NSAIDs, or unknown pharmacodynamics of the drug that increase the risk of adverse effects.^12^ A history of phenylbutazone administration was more frequent than any other NSAID in this study, likely due to more frequent usage in comparison to other NSAIDs.^12^ In Australia, oral preparations of flunixin paste are not widely available in comparison to oral preparations of phenylbutazone. A combination of phenylbutazone and the COX‐2 selective NSAID meloxicam, resulted in 1 RDC case, however no cases were administered COX‐2 selective NSAIDs alone. It is possible that COX‐2 selective NSAIDs have an increased safety profile, however the lack of cases in this study is likely due to the low rate of administration of COX‐2 selective NSAIDs in Australia. RDC can occur after excessive dosages of meloxicam, suggesting that COX‐2 selective NSAIDs can cause gastrointestinal lesions if administered inappropriately.^1^ Concurrent administration of more than 1 NSAID is known as “stacking” and can predispose horses to RDC.^17^, ^18^, ^19^ NSAIDs have an additive effect, therefore 2 NSAIDs co‐administered at recommended doses is equivalent to administering double the recommended dose of 1 NSAID.^17^, ^18^, ^19^ Three out of 35 cases in the study had more than 1 NSAID administered concurrently.

An exact toxic dose for each NSAID is difficult to define, due to vast differences in pharmacokinetics, individual sensitivity and hydration status. This emphasizes the importance of administering NSAIDs following recommended dosages and providing client education on the risks of unmonitored use. It is unknown if the degree of NSAID overdose is correlated with the severity of RDC and increased risk of death. Sixteen percent of horses with known NSAID administration (4/25) developed RDC when administered recommended dosages, suggesting that an overdose of NSAIDs is not a prerequisite to developing the disease.

Diarrhea, colic, and tachycardia were the most common clinical presentations on hospital admission. The presence of diarrhea in 69% of cases is higher in comparison to previous studies, which reported rates of 20% and 65%.^1^, ^7^ Colic was evident in 61% of cases and pyrexia in 33% of cases, consistent with a previous report.^1^ It is unknown whether tachycardia was attributable to pain, hypovolemia, or stress. Weight loss and ventral edema were not reviewed in this study, however previous reports suggest these are common clinical findings of horses diagnosed with RDC.^1^, ^6^

**FIGURE 4:**
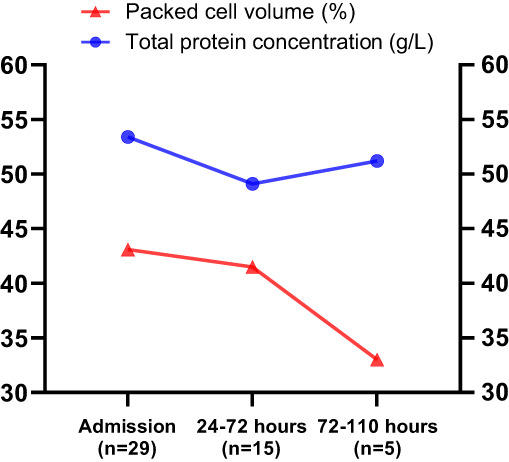
Mean PCV and TP values at admission, 24 to 72 hours after admission and 72 to 110 hours after admission.

Hypoalbuminemia, hypocalcaemia, and hyperlactatemia were the most common clinicopathological abnormalities. Hypoproteinemia was only evident in 50% of cases, which was lower than anticipated. Another study reported hypoproteinemia and hypoalbuminemia in 100% and 89% of cases respectively.^1^ The hematology and biochemistry results reported in the current study were sampled on hospital admission, therefore hematocrit and total protein concentrations could be relatively increased due to hemoconcentration. Figure 4 demonstrates a decrease in mean total protein concentrations 24 to 72 hours after hospital admission, likely due to administration of intravenous fluid therapy and rehydration in affected cases. However, only 60% (9/15) cases had hypoproteinemia 24 to 72 hours after admission, and at 72 to 110 hours after admission, only 50% (3/6) of cases reported hypoproteinemia which is still inconsistent with previous studies.^1^, ^7^, ^8^, ^9^ Hypoproteinemia and hypoalbuminemia occur due to intestinal protein loss, and hypocalcaemia is likely attributable to loss of protein‐bound calcium and reduced intake with inappetence. Ionized calcium concentration is a more appropriate measurement of physiologically active serum calcium, however was not routinely measured by institutions in this study.^20^

Right dorsal colitis has been associated with panhypoproteinemia, however to the best of our knowledge, serum globulin concentrations have not been reported in any previous case reports of RDC.^6^, ^16^ In the current study, hypoglobulinemia was reported in 41% of cases, compared with hypoalbuminemia in 83% of cases, suggesting that panhypoproteinemia is an inconsistent finding. Some cases demonstrated globulin concentrations in the upper interval of reference ranges and 1 case had hyperglobulinemia. Therefore, despite potential intestinal loss, globulins could be normal to increased likely due to antigenic stimulation of inflammation in the right dorsal colon. Normal globulin concentrations were reported in 16/27 (59%) cases and could have contributed to a lower number of cases with hypoproteinemia. This suggests that monitoring serum albumin concentration could be more sensitive than serum total protein in detecting early disease.

Anemia has been documented as a common clinical abnormality of RDC, however only 3/29 (10%) cases of decreased RBC concentration at hospital admission and 3/15 (20%) cases 24 to 72 hours post admission were found to be anemic in this study.^6^ Serum creatinine concentrations were increased in 24% of cases. Renal papillary necrosis is a potential sequelae to NSAID overdose and acute kidney injury and subsequent renal azotemia is also possible, therefore renal variables and urine specific gravity should be carefully monitored.^21^ One horse in the study had isosthenuria, increased creatinine and renal casts on admission, however these resolved before discharge. Another horse had renal papillary necrosis of the right kidney on post‐mortem examination. It is possible that more cases in the study could have had concurrent renal papillary necrosis. It is hypothesized that horses that are dehydrated before receiving NSAIDs are more predisposed to developing renal papillary necrosis in comparison to horses that receive NSAIDs while hydrated.^13^ A study found that all horses that received phenylbutazone and had concurrent water deprivation developed acute renal papillary necrosis, while horses that received phenylbutazone without water deprivation did not develop renal papillary necrosis.^21^ Horses with an increased heart rate, PCV, and abnormal mucous membranes at hospital presentation were found to have significantly decreased odds of survival. This suggests regular monitoring of serum albumin concentrations for horses receiving NSAIDs is warranted in attempt to detect RDC earlier in the course of disease.

Abdominal ultrasonography was performed in 31 cases. A total of 77% (24/31) cases reported subjective thickening of the right dorsal colon wall, with a median mural thickness of 10.0 mm (IQR 7‐13.5 mm). Normal mean wall thickness of the right dorsal colon in healthy ponies and horses is 2.7 ± 0.2 mm, and 3.7 ± 0.3 mm, respectively, therefore all 20 cases that provided measurements in this study had increased mural thickness of the right dorsal colon.^10^ Another study evaluated mural thickness of the right dorsal colon in horse diagnosed with RDC and reported values of 8.7 to 11.7 mm at the 13th and 11th intercostal space, consistent with our findings.^15^ Abdominal ultrasonography is considered to be a useful tool assisting in the diagnosis of RDC, however the procedure lacks sensitivity as it does not allow visualization of the mesenteric surface of the right dorsal colon.^1^, ^6^ In this study, 23% (7/31) of cases that underwent ultrasonography reported no subjective thickening of the right dorsal colon. This could be due to lack of appropriate visualization of the right dorsal colon, lack of operator experience, right dorsal colon lesions only present on the mesenteric surface of the colon or the examination being performed early in the course of the disease before thickening developed.

Treatment for RDC can involve medical and surgical management. In our study, 7/35 cases (20%) were managed surgically, with 2 of these cases requiring a colon resection. Of the 7 horses in which surgery was performed, 3 did not survive to discharge. Both cases requiring a colon resection did survive to discharge. Medical management encompasses eliminating NSAID use, altering the diet and use of medications. Misoprostol is a synthetic analogue of prostaglandin E_1_ which is thought to provide a protective effect to gastrointestinal mucosa by increasing mucus and bicarbonate secretion and enhancing mucosal blood flow.^22^ It has anti‐inflammatory effect on equine leukocytes in vitro.^23^ Misoprostol can be administered to RDC cases at dosages of 2 to 5 μg/kg every 6 to 12 hours.^6^ Although 16 horses were treated with misoprostol in our study, no statistically significant differences in survival was noted between medically treated cases that received misoprostol and those that did not. The lack of significant differences could have been due to low numbers administered misoprostol. Recommended dietary modification includes restricting the fiber in the diet for 3 to 6 months to reduce the mechanical and physiological load on the colon.^11^ Continuous feeding of psyllium husk is hypothesized to produce short‐chain fatty acids which can aid repair of colonic mucosa.^6^ Corn oil is a source of linoleic acid, which has been demonstrated to increase gastric prostaglandin E_2_ and promote mucosal healing.^24^ While no studies have evaluated the effect of corn oil for the repair of the colonic mucosa, it was included as part of the treatment plan in some cases.

Intravenous fluid therapy with crystalloids was provided in 20/34 cases (59%), likely to correct hypovolemia that might have been associated with diarrhea. It is likely that intravenous administration of fluids were associated with a lower rate of survival due to being administered to horses with a more severe presentation of the disease on hospital admission; 13/15 horses that did not survive to discharge received intravenous administration of fluid therapy. Antimicrobial therapy was used in 12/34 cases (35%) of cases, of which 3 had received peri‐operative antimicrobials. Metronidazole has anti‐inflammatory effects in experimental animals with intestinal injury and is administered to treat NSAID enteropathies in human medicine.^6^ It has been shown to inhibit leukocyte adherence to endothelial cells in mesenteric vessels in rats.^1^, ^8^ However, adequate antimicrobial stewardship does not support use of antimicrobials for potential anti‐inflammatory effects unrelated to antimicrobial activity.^25^ Omeprazole was administered in nearly half (47%) the cases to treat secondary gastric ulcers that might have been present. If there is a recent history of NSAID usage, it is recommended to delay administration of omeprazole due to increased risk of adverse intestinal events from co‐administration of NSAIDs and omeprazole.^26^ Prophylactic use of omeprazole in attempt to alleviate NSAID induced gastric ulcers is contraindicated, and omeprazole use should be reserved for horses with squamous gastric ulcers confirmed by gastroscopy.^26^ Sucralfate is a cytoprotective agent administered to treat duodenal and gastric ulcerations in horse and human patients. One study showed that co‐administration of phenylbutazone and sucralfate minimized the incidence of colonic ulcerations in foals in comparison to the phenylbutazone treatment alone, therefore could provide some benefit in treating RDC.^27^ In the current study, 38% (12/32) of horses received sucralfate.

Case fatality rates of RDC in the few reported retrospective studies are variable, case fatality rates of: 92% (12/13^8^), 20% (1/5^7^), 33% (1/3^9^), and 40% (25/63^1^). It is difficult to compare case fatality rates among the studies due to varying sample size, case definitions, severity, and treatments. The overall case fatality rate in this study was 43% (15/35). All studies have a lower case fatality rate than first reported as 92% in 1990,^8^ suggesting there has been substantial improvements in earlier detection and advancement in treatment of RDC. Improvements in human medicine for treating NSAID toxicosis have contributed to increased education of veterinarians in the recognition and treatment of the disease.^28^

## 5 LIMITATIONS

Being a retrospective case series, limitations to our study included missing or unknown data for some variables and a dependence on the accuracy of the medical records. There is potential bias in history of NSAID usage reported, with the possibility of owners inaccurately reporting doses of NSAID administered.^6^ A lack of homogeneity was evident in the study, with cases recorded from different institutions and clinicians. There was a lack of homogeneity in ultrasound machines and clinicians performing the procedures, and the intercostal space in which the mural thickness was recorded was often not documented. A definitive diagnosis was not reached in all cases where biopsy, necropsy or celiotomy was not performed, therefore some cases remained as a presumptive diagnosis of RDC based on a set of criteria. Differences in treatment plans, financial limitations of the owners, and varying criteria for discharge between institutions could have influenced variables such as survival rate and length of hospitalization.

## 6 CONCLUSION

Excessive dosages and prolonged administration of NSAIDs before the development of RDC was found in most cases, however RDC also developed when recommended dose rates were used. Horses that receive a prolonged course of NSAIDs should be vigilantly monitored utilizing serum albumin concentrations. Veterinarians should improve client education regarding the risks of inappropriate NSAID usage. Diarrhea, colic, and tachycardia are the most common clinical presentations, and NSAID administration should discontinue as soon as any clinical signs become apparent. Hypoalbuminemia, hypocalcemia, and hyperlactatemia are common clinicopathological findings of RDC. The presence of hypoproteinemia and panhypoproteinemia was less common than hypoalbuminemia. Factors associated with a decreased survival included an increased heart, PCV, and abnormal mucous membranes on hospital presentation. Abdominal ultrasonography is a useful diagnostic tool for RDC, however there is a lack of standardization and could lack sensitivity. The overall prognosis for RDC is improving, likely with greater awareness, earlier recognition, and aggressive treatment. The results of this study could assist veterinarians in forming a presumptive diagnosis of RDC, with earlier detection and treatment likely contributing to an increased survival rate for the disease.

## CONFLICT OF INTEREST DECLARATION

Authors declare no conflict of interest.

## OFF‐LABEL ANTIMICROBIAL DECLARATION

Authors declare no off‐label use of antimicrobials.

## INSTITUTIONAL ANIMAL CARE AND USE COMMITTEE (IACUC) OR OTHER APPROVAL DECLARATION

Authors declare no IACUC or other approval was needed.

## HUMAN ETHICS APPROVAL DECLARATION

Authors declare human ethics approval was not needed for this study.
